# Antioxidant Properties and Oxidative Transformation of Different Chromone Derivatives

**DOI:** 10.3390/molecules22040588

**Published:** 2017-04-06

**Authors:** Evelin Csepanyi, Peter Szabados-Furjesi, Attila Kiss-Szikszai, Lisa M. Frensemeier, Uwe Karst, Istvan Lekli, David D. Haines, Arpad Tosaki, Istvan Bak

**Affiliations:** 1 Department of Pharmacology, Faculty of Pharmacy, University of Debrecen, Debrecen H-4032, Hungary; csepanyi.evelin@pharm.unideb.hu (E.C.); szabados-furjesi.peter@pharm.unideb.hu (P.S.-F.); lekli.istvan@pharm.unideb.hu (I.L.); donald.david.haines@pharm.unideb.hu (D.D.H.); tosaki.arpad@pharm.unideb.hu (A.T.); 2 Department of Bioanalytical Chemistry, Faculty of Pharmacy, University of Debrecen, Debrecen H-4032, Hungary; 3 Department of Organic Chemistry, Faculty of Science and Technology, University of Debrecen, Debrecen H-4032, Hungary; kiss.attila@science.unideb.hu; 4 Institute of Inorganic and Analytical Chemistry, University of Muenster, Muenster D-48149, Germany; l_fren02@uni-muenster.de (L.M.F.); uk@uni-muenster.de (U.K.)

**Keywords:** antioxidants, chromone, flavonoids, cytotoxicity, oxidative metabolism

## Abstract

Nowadays, there is an increase in the application of natural products for the prevention of different disorders or adjuvant substances next to pharmacological treatment. Phytochemicals include different chromone derivatives, which possess a wide spectrum of biological activity. The aim of the present study was the investigation of the antioxidant activity, cytotoxicity and oxidative transformation of nine chromone derivatives. First, we investigated the radical scavenging activity (ABTS), the oxygen radical absorption capacity (ORAC) and the ferric reducing antioxidant power (FRAP) of the investigated molecules. The cytotoxic effects of the compounds were tested on H9c2 cell cultures by the MTT assay. Each compound showed a significant ORAC value compared to the reference. However, the compound 865 possess significantly higher FRAP and ABTS activity in comparison with the reference and other tested molecules, respectively. Based on these assays, the compound 865 was selected for further analysis. In these experiments, we investigated the oxidative metabolism of the compound in vitro. The molecule was oxidized by the Fenton reaction, artificial porphyrin and electrochemistry; then, the formed products were identified by mass spectrometry. Four possible metabolites were detected. The results revealed the compound 865 to possess good antioxidant properties and to be stable metabolically; hence, it is worth investigating its effects in vivo.

## 1. Introduction

Oxidative/nitrosative stress is a phenomenon that is related to the formation of free radicals, reactive oxygen and nitrogen species (ROS/RNS) in excess. One of the major consequences of oxidative stress is the damage of biological macromolecules, including DNA, membrane lipids and proteins, leading to cell death. The uncontrolled oxidation of these molecules contributes to the development and/or progression of many diseases, such as ischemic heart disease, cancer, diabetes and stroke.

In spite of the intensive research, the number of patients diagnosed with oxidative stress-related diseases is progressively increasing worldwide. Thus, the identification of novel countermeasures for oxidative stress-induced pathologies is a major priority in both basic and clinical research, with particular emphasis on drug discovery efforts focused on natural compounds with antioxidant properties. Many plants contain so-called phytochemicals, which possess significant antioxidant properties. Indeed, epidemiological data clearly demonstrate that persons with phytochemical-rich diets experience significantly lower incidence of chronic illness as a direct result of such foods mitigating the effects of oxidative stress. It has been shown that grape seed proanthocyanidins have cardio-, reno- and hepato-protective effects [1,2,3]. Other examples include work by the authors of the present report, showing cardioprotective effects of sour cherry seed kernel extract on ischemia/reperfusion-induced damage in isolated “working” diabetic and non-diabetic rat hearts [4,5]. Moreover, many laboratories worldwide have investigated the effects of *Ginkgo biloba* in different diseases [6,7,8,9]. Although, these examples show only a very narrow cross-section of the possible application of phytochemicals, there is a general agreement that the different natural antioxidant compounds, produced mainly by plants, exhibit huge promise as stand-alone or adjuvant agents in preventive medicine and therapy for diseases in which oxidative stress-induced tissue damage is a factor. Bioactive phytochemicals produced by plants prominently include phenoloids, terpenoids and alkaloids with antioxidant properties [10]. Among these, phenoloids are the largest group and comprise, among others, anthocyanins, coumarins (benzo-α-pyrones) and chromones (benzo-γ-pyrones). Flavonoids are the phenyl derivatives of chromones and possess well-known antioxidant activity. The antioxidant activity strongly depends on the substitution of the rings. The position and the type of the substituents are primary determinants of their biological properties [11].

The present investigation was conducted to evaluate the antioxidant properties and oxidative transformation of nine chromone derivatives (Table 1) selected from the molecule bank of the University of Debrecen. In our experiments, we determined the radical scavenging activity of the compounds using free radical scavenger assays. Furthermore, we measured the oxygen radical absorption capacity (ORAC) and the ferric reducing antioxidant power (FRAP) of the investigated compounds.

In additional studies, we determined the cytotoxicity on H9c2 cardiomyoblastoma cell cultures by the MTT assay and investigated the effects of the compounds against H_2_O_2_-induced cell death. Finally, following the selection of compounds according to their antioxidant properties, we investigated the oxidative transformation using biomimetic model systems including the Fenton reaction, artificial porphyrin and electrochemistry coupled to mass spectrometry (EC-MS) in order to obtain information about the metabolic stability.

## 2. Results

**Figure 1:**
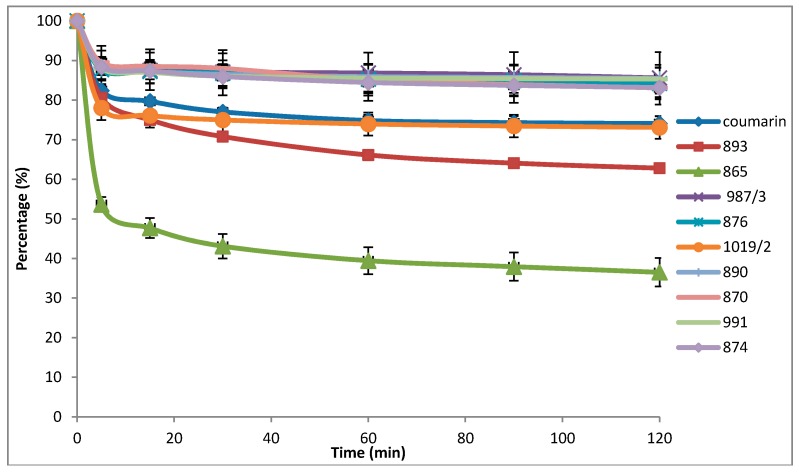
Free radical scavenger activity against the ABTS radical. All measurements were repeated three times. Data are expressed as the mean ± SEM.

In the first series of our experiments, we investigated the antioxidant properties of the compounds of interest. Results included negligible scavenging occurring in the case of galvinoxyl and DPPH radicals (data not shown), while compound Number 865, 4-*N*,*N*-dimethylamino-flavon (DMAF), exhibited significant scavenging activity against the ABTS radical (Figure 1).

**Figure 2:**
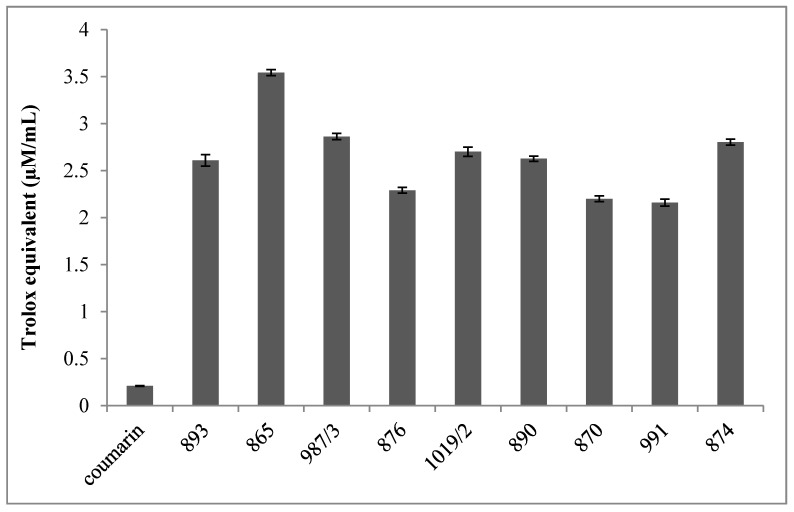
Oxygen radical absorption capacity of the tested molecules. All measurements were carried out in duplicate and repeated four times for each investigated compounds. Data are expressed as the mean ± SEM.

Furthermore, each compound has significantly higher oxygen radical absorption capacity (ORAC) compared to coumarin, which was here used as the reference standard (Figure 2). Moreover, DMAF had the highest ORAC value.

**Figure 3:**
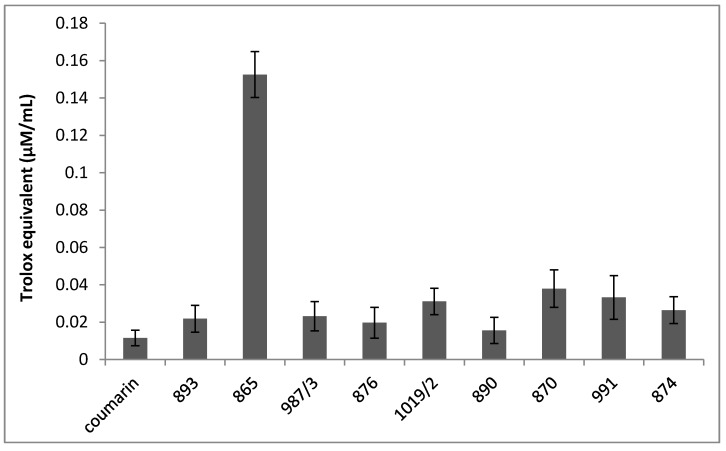
Ferric reducing antioxidant power of the investigated chromone derivatives. All measurements were carried out in duplicate and repeated four times for each investigated compounds. Data are expressed as the mean ± SEM.

Figure 3 shows the ferric reducing antioxidant power (FRAP) of the investigated chromone derivatives; and it was observed that some of the compounds (893, 987/3, 876 and 890) exhibited FRAP values similar to coumarin, while others, such as 1019/2, 870, 991 and 874, possess a slightly increased trolox equivalent. Moreover, in the case of DMAF, significantly increased FRAP activity was noted in comparison to the reference standard (coumarin) or the other compounds tested.

**Figure 4:**
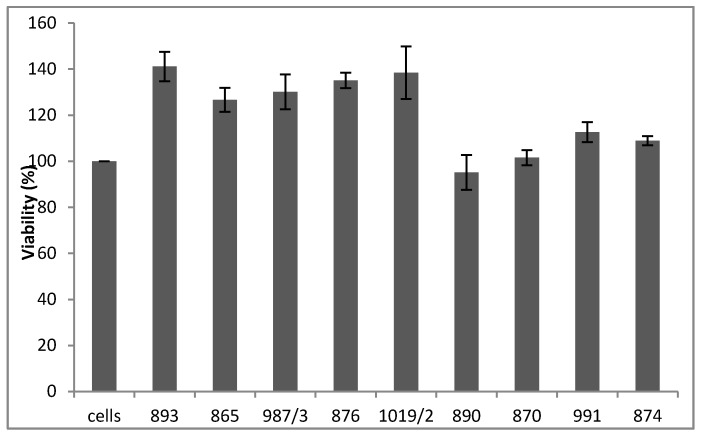

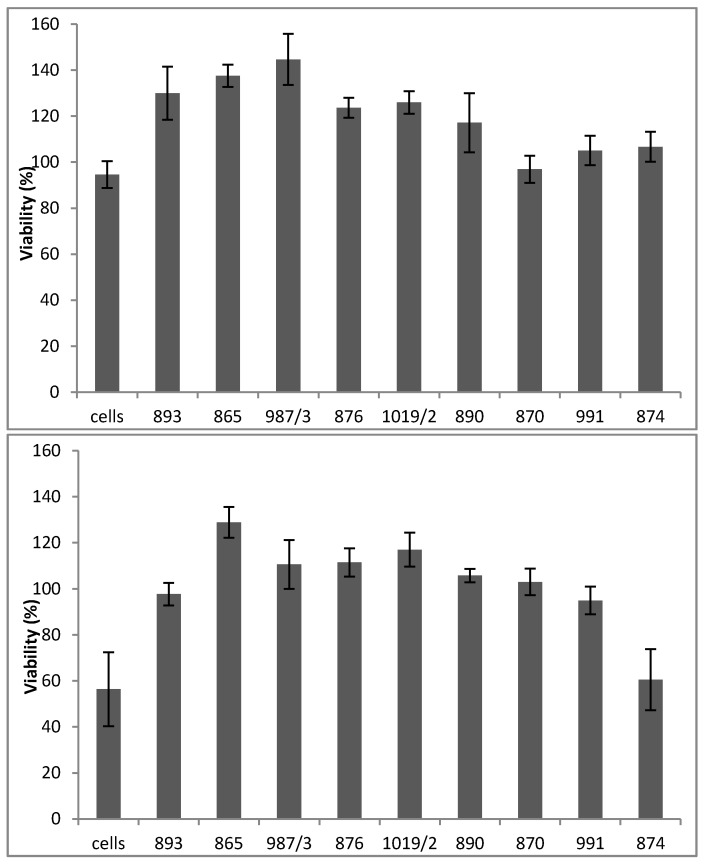
Cytotoxicity (**upper panel**) and the effects on H_2_O_2_-induced cell death (**middle** and **lower panels**) of the test compounds measured by MTT. Absorbance values were averaged across four replicate wells and repeated three times. Data are expressed as the mean ± SEM.

The results of the MTT assay in Figure 4 reveal that none of the tested compounds mediated the cytotoxic effect (Figure 4, upper panel). Furthermore, six out of the nine molecules (893, 865, 987/3, 876, 1019/2 and 890) significantly augmented H9c2 cell viability when the cells were treated with 125 (Figure 4, middle panel) or 250 (Figure 4, lower panel) μM H_2_O_2_, respectively.

Based on the beneficial properties of DMAF demonstrated by the antioxidant assays, this compound was investigated more closely towards its oxidative stability using biomimetic model systems. Initially, the oxidation of DMAF was performed by the chemical Fenton reaction followed by an off-line ESI-MS analysis (spectra not shown) in order to obtain information on the oxidation behaviour of the compound. By this means, three possible metabolites were detected with *m*/*z* 252.1, *m*/*z* 238.1 and *m*/*z* 282.1. Based on the peak intensities, the oxidation product with *m*/*z* 252.1 was formed with the highest yield, then 282.1, and with the lowest yield, the 238.1 oxidative product was obtained.

In additional experiments, we investigated the oxidative transformation of DMAF by the application of Fe(III) meso-tetra (4-sulfonatophenyl) porphine chloride. The metabolites were detected by HPLC-MS/MS (spectra not shown). Based on the analysis of the measured spectra, the oxidation products with *m*/*z* 238.1 and *m*/*z* 282.1 were also detected. Furthermore, other possible metabolic products with *m*/*z* 280.1 and *m*/*z* 265.5 were also detected. The oxidation product with *m*/*z* 252.1 was additionally detected; however, it was also present in the control sample with the same retention time as in the sample and is thus most likely an impurity of DMAF. In this case, the major product was found to be *m*/*z* 280.1.

**Figure 5:**
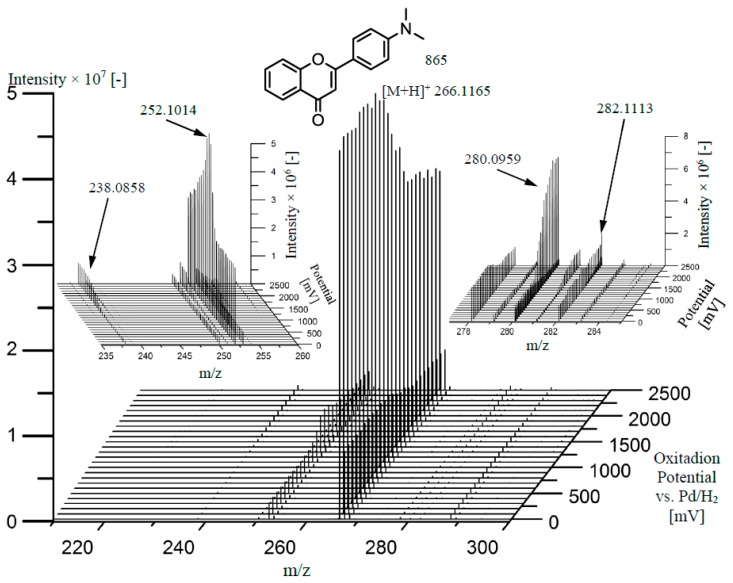
Electrochemical oxidation of 4-*N*,*N*-dimethylamino-flavon (DMAF) by potential ramp application of 0–2500 mV.

Finally, the oxidation of DMAF was carried out using an electrochemical cell coupled to mass spectrometry. Therefore, a potential ramp was applied to the EC cell while mass spectra where recorded continuously. Plotting the mass spectra in dependence of the applied potential, a so-called mass voltammogram was obtained (Figure 5). The oxidation of DMAF (*m*/*z* 266.1165) can be identified by the decreasing signal intensity at increasing potentials starting at around 1500 mV. Moreover, *m*/*z* 252.1014 was detected already without potential application, which most likely can be attributed to an ion source oxidation. Nonetheless, the signal intensity of *m*/*z* 252.1014 increases at higher potentials representing the electrochemical oxidation, but afterwards also decreases, indicating a further oxidation of this product. With similarity to the Fenton system, the major oxidation product was found to be *m*/*z* 252.1014, while the product with *m*/*z* 280.0959, which was also produced by the artificial porphyrin, was generated with the second highest yield. Using EC-MS, the other metabolites with *m*/*z* 238.0858 and *m*/*z* 282.1113 could also be detected (Figure 5, insert panels).

## 3. Discussion

Phytochemicals are compounds occurring at high concentrations mainly in vegetables, fruits and their seeds and legumes. These are not essential micronutrients, but possess significant health-promoting properties. The main classes of these are alkaloids, terpenoids and phenoloids. Among phenolids, many molecules have a chromone (4*H*-benzopyran-4-one) or a coumarin (2*H*-benzopyran-2-one) basic skeleton. Both compound types possess a wide range of biological properties, including antioxidant, anti-inflammatory, antimicrobial, antiallergenic, anti-ischemic, antiviral, anti-hypertensive and antitumor activities [12,13]. Because the isolation and purification is not efficient and generally results in low yield of the bioactive compounds, many laboratories worldwide make an effort in the synthesis or semisynthetic modification of different compounds with natural origin in order to obtain derivatives with enhanced biological activities and prepare these at large scale, which is suitable to carry out in-depth pharmacological studies in vitro and in vivo. Certain phytochemical classes have molecular structural features that make them particularly suitable for the creation of derivatives with novel properties. For example, the coumarin and chromone scaffolds are promising ring systems for the design and synthesis of new molecules with different biological effects [14,15,16,17,18,19]. In this study, we made attempts to investigate the in vitro antioxidant activity and oxidative transformation of nine different chromone derivatives. For the characterization of the antioxidant properties, either electron transfer (ET)- or hydrogen atom transfer (HAT)-based assays were chosen as the main investigative tools. In the case of the HAT-based assay (ORAC), each compound showed significant activity, with DMAF (865) manifesting a significantly higher ORAC value in comparison with phytochemicals based on other flavonoid structures. Surprisingly, the results of the ET-based assays revealed that DMAF, which is a simple flavonoid derivative, has the most robust FRAP and radical scavenger activity against the ABTS radical. It is thus noteworthy that in different studies when ET-based (ABTS, DPPH, FRAP) assays were used to compare antioxidant activity, the results were similar, while for those in which the ORAC test was used, the results were different. It is also important to note that on the basis of a single test, a compound cannot be definitively classified as an antioxidant; hence, a series of tests to make this determination is typically necessary. Another factor to consider is that the values of the assays used to determine the antioxidant activity of a particular compound may vary from study to study despite testing standardization by investigators. This problem poses challenges in the unified interpretation of assay outcomes for new compounds in the context of earlier reported data. Based on our results, DMAF exhibited significant antioxidant activity in each assay with no significant cytotoxicity. Moreover, DMAF also prevented H_2_O_2_-induced cell death, a property that underscored its potential for future studies of its metabolic stability and possible benefits to human and veterinary health.

**Figure 6:**
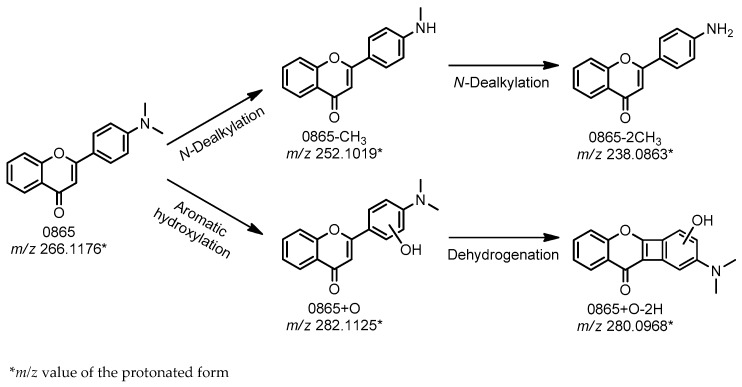
Proposed metabolic transformation of DMAF.

The above-mentioned factors notwithstanding, the positive outcomes of the assessment of the antioxidant effects under in vitro conditions are not typically considered to be an independently sufficient cause for the allocation of time and resources for a particular phytochemical compound as a candidate for the further stages of the drug R&D process. Other properties of the molecule of interest must be considered, such as the stability and toxicity of the compound. One of the most important factors that determines the toxicity and pharmacologic effects of a drug candidate is its oxidative metabolism. Therefore, it is very important to investigate the metabolic stability of the investigated compounds. The so-called biomimetic systems may be used as an essential component of preclinical studies to select the most suitable drug candidates for later extended pharmacokinetic and toxicological animal tests and eventually for clinical (phases I–III) studies. Oxidation can be performed either by the Fenton reaction, systems containing synthetic porphyrins or by the application of electrochemistry using either off-line or on-line mass spectrometric detection [20]. The Fenton system was shown to be capable of stimulating *N*-dealkylation, *S*-oxidation and the hydroxylation reaction; metalloporphyrins are most suitable to mimic *S*-oxidation, *N*-dealkylation, *N*-oxide formation, epoxidation, hydroxylation and dehydrogenation, while EC-triggered systems are suitable for modelling *N*-dealkylation, *S-*, *P*-oxidation, aromatic hydroxylation and dehydrogenation reactions [21]. By use of each of these three assays in the present study, four possible metabolites of DMAF were detected. The corresponding proposed reaction scheme is illustrated in Figure 6.

First, *N*-dealkylation of a tertiary amine to a secondary amine (*m*/*z* 252.1019) was obtained, followed by the removal of a further methyl group, resulting in a primer amine (*m*/*z* 238.0863). These products were detected by means of all three model systems (Table 2) since each technique is suitable for mimicking *N*-dealkylation reactions. Additionally, the formation of the product with *m*/*z* 282.1125 presumably resulted from processes that include aromatic hydroxylation on the B ring, a conclusion that was deduced from the observed fragmentation pattern of this molecule (data not shown). The following dehydrogenation leading to *m*/*z* 280.0968 though a (possible) cyclization between the C3 and C6′ is proposed. Because the applied methods constitute a good model system for hydroxylation, it is not surprising that this product was detected in each case. The dehydrogenated form was not detected by the Fenton reaction, possibly because of the low concentration of this product, which may have been at levels below the limit of detection.

## 4. Materials and Methods

### 4.1. Chemicals

Water (ultra-pure) was prepared with the SolPure 78 water purification system from Poll Lab (Bielsko-Biala, Poland). Ethylenediaminetetraacetic acid disodium salt dihydrate (EDTA-Na_2_), L(+)-ascorbic acid, ethanol (96%), acetic acid and formic acid were obtained from Scharlab Magyarország Kft. (Debrecen, Hungary). Iron(III) chloride, hydrogen peroxide solution, potassium hydrogen phosphate, monopotassium phosphate, sodium citrate, citric acid, 2,2′-azobis (2-amidinopropane) dihydrochloride (AAPH), sodium acetate trihydrate, 2,4,6-Tris(2-pyridyl)-*s*-triazine (TPTZ), 2,2′-azino-bis(3-ethylbenzothiazoline-6-sulfonic acid) diammonium salt (ABTS-salt), 3-(4,5-dimethylthiazol-2-yl)-2,5-diphenyltetrazolium bromide (MTT), isopropanol and fluorescein were obtained from Sigma-Aldrich Kft. (Budapest, Hungary). Fe(III) meso-tetra(4-sulfonatophenyl)porphine chloride was obtained from Frontier Scientific (Logan, UT, USA). Ammonium formate (≥99.995%, trace metal basis) was ordered from Sigma-Aldrich (Steinheim, Germany). Acetonitrile (LC-MS grade) was delivered from Merck (Darmstadt, Germany).

Test compounds were selected from the molecule bank of the University of Debrecen. This compound “library” contains nearly 3000 compounds available for investigative use at high purity (>95%), including a large number of oxygen-containing heterocyclic compounds along with their precursors. Among these compounds, 175 bear the flavonoid moiety. Test compounds were randomly selected from this subset for possible use in the present investigation. A major criterion in this selection process, was novelty; specifically, phytochemicals were chosen that had never been previously subjected to the kind of analyses conducted in the experiments described in the present report. Another major strategy in this selection process is that the range of compounds chosen represented the greatest extent of the entire substitution pattern for the flavonoid ring within the constraint of the choices offered by this small library.

### 4.2. ABTS Assay

The ABTS solution was prepared by dissolving ABTS-salt (2,2′-azino-bis(3-ethylbenzothiazoline-6-sulfonic acid) diammonium salt) in citric acid-sodium citrate buffer (pH 6, 50 mM) containing 2% ethanol (96%). ABTS**^+^** (100 µM) was generated with the ABTS/H_2_O_2_/metmyoglobin system and mixed with the investigated compounds (25 µM). The reaction was carried out for 2 h at room temperature. During this period, the absorbance of ABTS^+^ was monitored at 730 nm with a Helios-α-spectrophotometer at 0, 5, 15, 30, 60, 90, 120 min. All measurements were repeated three times. Data are expressed as the mean ± SEM. The results can be seen in Figure 1.

### 4.3. ORAC Assay

The ORAC assay was carried out as described by Glazer et al. [22], with modification. Freshly-prepared AAPH stock (37.5 mM) solution and fluorescein stock solution (0.6 µM) in phosphate buffer 75 mM, (pH 7.0) were used for the assay. The concentration of the investigated compounds was 0.05 mg/mL. For all experiments, the final reaction volume of the wells was 200 µL, containing 65 µL of buffer, 20 µL of fluorescein, 15 µL of compounds of interest, except the blank and the reactions were initiated by an addition 100 µL of AAPH stock solution. Black-sided micro plates were incubated at 37 °C. The fluorescence (excitation wavelength of 485 nm and emission wavelength of 520 nm) was monitored every 2 min for 1 h by the FLUOstar OPTIMA (BMG LABTECH) plate reader. The area under the curve (AUC) was calculated as the following:

AUC= 0.5 + (R2/R1) + (R3/R1) + (R4/R1) + …. + (Rn/R1)
(1)

where R1 is the initial fluorescence reading at 0 min and Rn is the fluorescence reading at n min. Finally, the net AUC was obtained by subtracting the AUC of the blank from that of a sample.

AUCnet = AUC sample − AUC blank
(2)

All measurements were carried out in duplicate and repeated four times for each tested compound. Data are expressed as the mean ± SEM. Results are shown in Figure 2.

### 4.4. FRAP Assay

The ferric reducing ability of the investigated compounds was determined by the FRAP assay. The prepared stock solutions for the assay contained acetate buffer solution (pH 3.6, 300 mM), TPTZ solution (10 mM) in 40 mM HCl and ferric chloride (FeCl_3_) solution (20 mM). The freshly-prepared working solution contained 25 mL of acetate buffer and 2.5–2.5 mL of TPTZ and ferric chloride solution, and the FRAP reagent was activated by incubation on 37 °C for 15 min. The reaction mixture consisted of 950 µL FRAP working solution and 50 µL of the investigated compound (0.05 mg/mL), except the blank sample. After 15 min of incubation at 37 °C, the absorbance of the coloured product was measured at 593 nm with a Helios-α-spectrophotometer. The FRAP values were expressed as trolox equivalents (µM/mL), based on the trolox standard calibration curve prepared by measuring of the different concentrations of trolox samples (0.015, 0.045, 0.105 and 0.21 mM). All measurements were carried out in duplicate and repeated four times for each investigated compounds. Data are expressed as the mean ± SEM. Results can be found in Figure 3.

### 4.5. Measurement of Cytotoxicity

Evaluation of the cytotoxicity of the investigated compounds on cellular survival was accomplished using the 3-(4,5-dimethylthiazol-2-yl)-2,5-diphenyltetrazolium bromide (MTT) assay. Briefly, H9c2 cells (ATCC, CRL-1446, LGC Standards GmbH, Wesel, Germany) dissociated by trituration in medium (Dulbecco’s modified eagle’s medium from Sigma with 10% FBS, 1% penicillin-streptomycin) were seeded into 96-well plates at a density of 3000 cells/well and cultured for 1 day to establish adhesion of the wells. Next day cells were treated with 150 µM antioxidant containing medium. Following a 30-min incubation period, the wells were treated with 0, 125 or 250 µM H_2_O_2_. Four hours later, the addition of 20 µL MTT solution (5 mg/mL in PBS) to each well and an additional 3 h incubation at 37 °C to allow mitochondrial uptake was performed. Finally, the medium was removed, and cells were lysed by the addition of 150 µL of isopropanol, incubated for 15 min followed by the measurement of absorbance at 570 and 690 nm using a plate reader (FLUOstar OPTIMA, BMG Labtech). Within each experiment, the absorbance values were averaged across 4 replicate wells and repeated 3 times. The cytotoxic effect assessments were estimated based on the linear correlation of the absorbance values with MTT-associated H9c2 viability and reported as the percentage of cells surviving 4 h of investigated antioxidant exposure relative to control cells not exposed to compounds. Data are expressed as the mean ± SEM. The results can be seen in Figure 4.

### 4.6. The Chemical Fenton System

The chemical Fenton system was prepared based on a method reported previously by Jurva et al. [23]. The reaction mixture consisted of 400 μL 2.5 mM 865 in acetonitrile, 50 μL 20 mM FeCl_3_ and 50 μL 20 mM EDTA-Na_2_ in acetonitrile/water (50:50), 500 μL 10 mM ascorbic acid in acetonitrile/water (50:50) and 1 μL 30% hydrogen peroxide. The control sample in the absence of hydrogen peroxide and the blank sample without the test compound were prepared. The mixtures were stirred at room temperature at 600 rpm. Samples were drawn at 30 min, 140 min, 18 h and 261 h and were analysed immediately by an API 2000 Triple Quadrupole mass spectrometer (Applied Biosystems, Waltham, MA, USA) equipped with a syringe pump. The flow rate was set to 100 μL/min, curtain gas 10 PSI, declustering potential 20 V, ion spray voltage 4000 V, focusing potential 400 V, ion source temperature 200 °C. ESI mass spectra were recorded in the range of *m*/*z* 100–500 in positive-ion mode with Analyst 1.5.1. Software (AB SCIEX, Concord, ON, Canada).

### 4.7. Oxidation by Artificial Porphyrin

The porphyrin system was prepared based on a method reported previously by Johansson et al. [21]. The reaction mixture consisted of 50 μL 10 mM 865 in acetonitrile, 35 μL acetonitrile, 315 μL 100 mM formic acid, 50 μL 10 mM Fe(III) meso-tetra(4-sulfonatophenyl)porphine chloride and 50 μL 30% H_2_O_2_. The control sample in the absence of hydrogen peroxide and the blank sample without the test compound were prepared. The mixtures were shaken at 37 °C for 30 min at 700 rpm in a shaking water bath. One hundred microlitres of the reaction mixture were diluted to 2 mL with acetonitrile/water (50:50). After HPLC separation (column: Kinetex XB-C18 2.6 μm, eluent: 0.1% formic acid in water and acetonitrile with 0.1% formic acid, gradient elution), the samples were introduced into an LTQ XL linear ion trap mass spectrometer (Thermo Fisher Scientific, Waltham, MA, USA) under the following conditions: sheath gas flow rate 35.00 a.u., spray voltage 5000 V, capillary temperature 300 °C, capillary voltage 31 V, tube lens voltage 150 V, skimmer voltage 34 V.

### 4.8. Electrochemical Oxidation

*N*,*N*-Dimethylaminoflavone (DMAF) was oxidized using an electrochemical thin-layer cell (FlexCell, Antec, Zoeterwoude, The Netherlands). The cell was equipped with a boron-doped diamond (BDD) working electrode, a graphite doped Teflon counter electrode and a Pd/H_2_ reference electrode. A potential ramp from 0–2500 mV with a scan rate of 10 mV/s was applied using a homemade potentiostat. DMAF in ammonium-formate (10 mM, pH 7.4) and acetonitrile 50/50 (*v*/*v*) was continuously pumped through the cell with a flow-rate of 10 µL/min delivered by a syringe pump (Model 74900, Cole Parmer, Vernon Hills, IL, USA). The effluent of the cell was introduced into an Exactive mass spectrometer (Thermo Fisher Scientific, Bremen, Germany). The full scan spectra (*m*/*z* 100–800) were recorded in ESI(+) mode under the following conditions: sheath gas flow rate 10.00 a.u., spray voltage 4000 V, capillary temperature 250 °C, capillary voltage 42.5 V, tube lens voltage 135 V, skimmer voltage 34 V. The software XCalibur 2.1 (Thermo Fisher Scientific, Bremen, Germany) was applied for data processing, and data visualization was performed by plotting the recorded mass spectra in dependence of the applied potential in the form of a three-dimensional mass voltammogram using the software Origin 9.1 (OriginLab, Northhampton, MA, USA). Results are shown in Figure 5.

## 5. Conclusions

The major value of the present study is in offering a full characterization of the phytochemical antioxidant ability for potential use in health maintenance provided by the data shown; and also, the strategy for producing this. A particularly significant result in this context is the observation that 4-*N*,*N*-dimethyl-flavone exhibited significant antioxidant activity and was metabolically stable. This outcome thus justifies its use as a research tool in the investigation of its effects in selected animal models of oxidative stress-related disease. Ongoing characterization of these processes is continuing by this laboratory. Furthermore, each compound evaluated here demonstrated significant ORAC activity, along with supporting evidence of their antioxidant capacities as assessed and further characterization by TRAP, CUPRAC, SOD and catalase activity, and the effects on the mitochondrial e^−^ transport chain, by our laboratory and other investigators, demonstrate significant potential for return-on-investment (ROI) in the design of novel strategies for human and animal use for phytochemical antioxidants.

## Figures and Tables

**Table 1:**
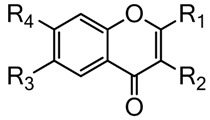
Compounds investigated in the study.

ID Number	R_1_	R_2_	R_3_	R_4_
893	3,4-dihydro-2*H*-1,5-benzodioxepin-7-yl	H	H	OH
865	4-(dimethylamino)phenyl	H	H	H
987/3	3,4-dihydro-2*H*-1,5-benzodioxepin-7-yl	H	Me	Me
876	2*H*-1,3-benzodioxol-5-yl	H	H	H
1019/2	phenyl	OAc	H	H
890	phenyl	H	NAc	H
870	4-bromophenyl	H	H	H
991	4-methoxyphenyl	H	Me	Me
874	4-[(4-methylpiperazin-1-yl)carbonyl]phenyl	H	H	H

**Table 2:** Detected possible metabolites of DMAF.

Product	Fenton System	EC System	Porphyrin System
865 − CH_3_	**+**	**+**	− *
865 − 2CH_3_	**+**	**+**	**+**
865 + O	**+**	**+**	**+**
865 + O − 2H	**−**	**+**	**+**

* It was detected, however considered as an impurity.

## References

[1] Pataki T., Bak I., Kovacs P., Bagchi D., Das D.K., Tosaki A. (2002). Grape seed proanthocyanidins improved cardiac recovery during reperfusion after ischemia in isolated rat hearts. Am. J. Clin. Nutr..

[2] Bao L., Zhang Z., Dai X., Ding Y., Jiang Y., Li Y., Li Y. (2015). Effects of grape seed proanthocyanidin extract on renal injury in type 2 diabetic rats. Mol. Med. Rep..

[3] Xu Z.C., Yin J., Zhou B., Liu Y.T., Yu Y., Li G.Q. (2015). Grape seed proanthocyanidin protects liver against ischemia/reperfusion injury by attenuating endoplasmic reticulum stress. World J. Gastroenterol..

[4] Bak I., Lekli I., Juhasz B., Nagy N., Varga E., Varadi J., Gesztelyi R., Szabo G., Szendrei L., Bacskay I. (2006). Cardioprotective mechanisms of Prunus cerasus (sour cherry) seed extract against ischemia-reperfusion-induced damage in isolated rat hearts. Am. J. Physiol. Heart Circ. Physiol..

[5] Czompa A., Gyongyosi A., Czegledi A., Csepanyi E., Bak I., Haines D.D., Tosaki A., Lekli I. (2014). Cardioprotection afforded by sour cherry seed kernel: The role of heme oxygenase-1. J. Cardiovasc. Pharmacol..

[6] Zhou X., Qi Y., Chen T. (2017). Long-term pre-treatment of antioxidant Ginkgo biloba extract EGb-761 attenuates cerebral-ischemia-induced neuronal damage in aged mice. Biomed. Pharmacother..

[7] Wang Z., Zhang J., Ren T., Dong Z. (2016). Targeted metabolomic profiling of cardioprotective effect of Ginkgo biloba L. extract on myocardial ischemia in rats. Phytomed. Int. J. Phytother. Phytopharm..

[8] Haines D.D., Varga B., Bak I., Juhasz B., Mahmoud F.F., Kalantari H., Gesztelyi R., Lekli I., Czompa A., Tosaki A. (2011). Summative interaction between astaxanthin, Ginkgo biloba extract (EGb761) and vitamin C in suppression of respiratory inflammation: A comparison with ibuprofen. Phytother. Res. PTR.

[9] Haines D.D., Bak I., Ferdinandy P., Mahmoud F.F., Al-Harbi S.A., Blasig I.E., Tosaki A. (2000). Cardioprotective effects of the calcineurin inhibitor FK506 and the PAF receptor antagonist and free radical scavenger, EGb 761, in isolated ischemic/reperfused rat hearts. J. Cardiovasc. Pharmacol..

[10] Laher I. (2014). Systems Biology of Free Radicals and Antioxidants.

[11] Bubols G.B., Vianna Dda R., Medina-Remon A., von Poser G., Lamuela-Raventos R.M., Eifler-Lima V.L., Garcia S.C. (2013). The antioxidant activity of coumarins and flavonoids. Mini Rev. Med. Chem..

[12] Machado N.F.L., Marques M.P.M. (2010). Bioactive chromone derivatives—Structural diversity. Curr. Bioact. Compd..

[13] Costa M., Dias T.A., Brito A., Proenca F. (2016). Biological importance of structurally diversified chromenes. Eur. J. Med. Chem..

[14] Balabani A., Hadjipavlou-Litina D.J., Litinas K.E., Mainou M., Tsironi C.C., Vronteli A. (2011). Synthesis and biological evaluation of (2,5-dihydro-1*H*-pyrrol-1-yl)-2*H*-chromen-2-ones as free radical scavengers. Eur. J. Med. Chem..

[15] Mladenovic M., Mihailovic M., Bogojevic D., Matic S., Niciforovic N., Mihailovic V., Vukovic N., Sukdolak S., Solujic S. (2011). In vitro antioxidant activity of selected 4-hydroxy-chromene-2-one derivatives-SAR, QSAR and DFT studies. Int. J. Mol. Sci..

[16] Takao K., Ishikawa R., Sugita Y. (2014). Synthesis and biological evaluation of 3-styrylchromone derivatives as free radical scavengers and alpha-glucosidase inhibitors. Chem. Pharm. Bull..

[17] Silva C.F., Pinto D.C., Silva A.M. (2016). Chromones: A Promising Ring System for New Anti-inflammatory Drugs. Chem. Med. Chem..

[18] Roma G., Braccio M.D., Carrieri A., Grossi G., Leoncini G., Grazia Signorello M., Carotti A. (2003). Coumarin, chromone, and 4(3*H*)-pyrimidinone novel bicyclic and tricyclic derivatives as antiplatelet agents: Synthesis, biological evaluation, and comparative molecular field analysis. Bioorgan. Med. Chem..

[19] Thanigaimalai P., Le Hoang T.A., Lee K.C., Sharma V.K., Bang S.C., Yun J.H., Roh E., Kim Y., Jung S.H. (2010). Synthesis and evaluation of novel chromone analogs for their inhibitory activity against interleukin-5. Eur. J. Med. Chem..

[20] Lohmann W., Karst U. (2008). Biomimetic modeling of oxidative drug metabolism: Strategies, advantages and limitations. Anal. Bioanal. Chem..

[21] Johansson T., Weidolf L., Jurva U. (2007). Mimicry of phase I drug metabolism—Novel methods for metabolite characterization and synthesis. Rapid Commun. Mass Spectrom. RCM.

[22] Glazer A.N. (1990). Phycoerythrin fluorescence-based assay for reactive oxygen species. Methods Enzymol..

[23] Jurva U., Wikstrom H.V., Bruins A.P. (2002). Electrochemically assisted Fenton reaction: Reaction of hydroxyl radicals with xenobiotics followed by on-line analysis with high-performance liquid chromatography/tandem mass spectrometry. Rapid Commun. Mass Spectrom. RCM.

